# Anticancer Drug Conjugates Incorporating Estrogen Receptor Ligands

**DOI:** 10.3390/pharmaceutics15010067

**Published:** 2022-12-26

**Authors:** Darius P. Zlotos, Thales Kronenberger, Stefan A. Laufer

**Affiliations:** 1 Department of Pharmaceutical Chemistry, Faculty of Pharmacy and Biotechnology, The German University in Cairo, New Cairo City 11835, Cairo, Egypt; 2 Institute of Pharmaceutical Sciences, Eberhard Karls Universität Tübingen, 72076 Tübingen, Germany; 3 Department of Internal Medicine VIII, University Hospital of Tübingen, 72076 Tübingen, Germany; 4 Cluster of Excellence iFIT (EXC 2180) ‘Image-Guided & Functionally Instructed Tumor Therapies’, University of Tübingen, 72076 Tübingen, Germany; 5 Tübingen Center for Academic Drug Discovery, Auf der Morgenstelle 8, 72076 Tübingen, Germany

**Keywords:** anticancer drug conjugates, estrogen receptor ligands, tumor targeting

## Abstract

Hormone-dependent cancers, such as certain types of breast cancer are characterized by over-expression of estrogen receptors (ERs). Anticancer drug conjugates combining ER ligands with other classes of anticancer agents may not only benefit from dual action at both anti-cancer targets but also from selective delivery of cytotoxic agents to ER-positive tumor cells resulting in less toxicity and adverse effects. Moreover, they could also take advantage of overcoming resistance typical for anti-hormonal monotherapy such as tamoxifen. In this review, we discuss the design, structures and pharmacological effects of numerous series of drug conjugates containing ER ligands such as selective ER modulators (tamoxifen, 4-hydroxytamoxifen, endoxifen), selective ER degraders (ICI-164384) and ER agonists (estradiol) linked to diverse anti-cancer agents including histone-deacetylase inhibitors, DNA-alkylating agents, antimitotic agents and epidermal growth factor receptor inhibitors.

## 1. Introduction

Anticancer drug conjugates (hybrid anticancer agents) are an emerging approach to overcome drawbacks of current anticancer treatment, such as insufficient potency and efficacy, high toxicity, and development of resistance [1,2]. Anticancer drug conjugates incorporate two drugs (pharmacophores) in one molecule exerting synergistic action at two different cancer targets [3]. Compared to a combination of two single-target drugs, a drug conjugate may offer the advantage of pharmacokinetic simplicity and fewer drug–drug interactions [4]. Drug conjugates should be distinguished from antibody⁠–drug conjugates (ADCs) which are monoclonal antibodies conjugated with cytotoxic small molecules. While ADCs have been successfully introduced as cancer therapeutics [5], hybrid anticancer agents have mostly been reported in preclinical studies [4]. However, the number of reports in the field has constantly increased in the last two decades. A PubMed data search using the term “anticancer hybrids” revealed the number of relevant publications to have risen from 33 in 1996 to 464 in 2021 [6]. Examples of anticancer drug conjugates that reached phase I clinical trials are CUDC-101, a dually acting chimeric EGFR/HDAC inhibitor derived from erlotinib and vorinostat [7,8], and CUDC-907, a dual HDAC-PI3K inhibitor [9].

**Figure 1:**
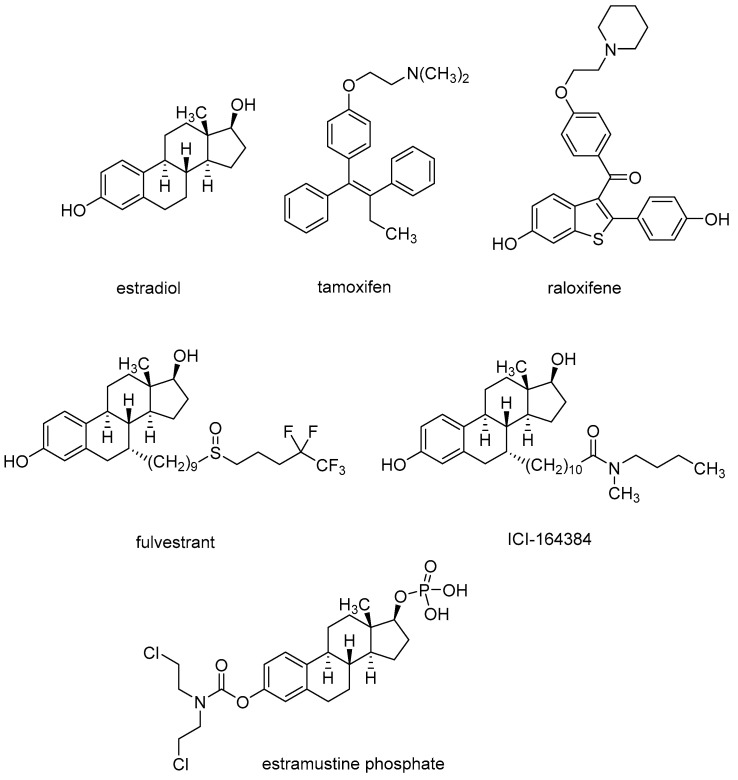
Structures of estrogen receptor ligands and estramustine phosphate.

Most anticancer drug conjugates target breast cancer, as recently reviewed [10]. Breast cancer is the most common type of neoplasia among women [11]. Estrogens, such as 17β-estradiol (Figure 1) are essential for the development of female sexual features including breast growth [12]. Estrogen action is mediated by intracellular estrogen receptors alpha and beta (ERα and Erβ) which belong to the superfamily of nuclear receptors. They regulate gene expression by binding to DNA response elements associated with target genes [13]. While ERα is expressed at low levels in normal tissues, it is overexpressed in the majority of hormone-dependent tumors that represent 75% of breast cancer [14]. Inhibition of estrogenic stimulation of ERα is therefore a widely used strategy in the pharmacotherapy of breast cancer [15]. Two classes of ER inhibitors are currently approved for breast cancer treatment, selective estrogen receptor modulators (SERMs), exemplified by tamoxifen and raloxifene, and selective estrogen receptor downregulators (SERDs), such as fulvestrant and ICI-164384. While SERMs show either antagonist or (partial) agonist action dependent on tissues (e.g., tamoxifen is an antagonist in breast cancer cells but has estrogenic effects on the uterus and bones), SERDs are pure anti-estrogens acting as antagonists in all tissues. Moreover, SERDs are known to induce ubiquitination and degradation of ERα through the proteasome pathway [16].

Estrogen receptor ligands have been often incorporated in anticancer hybrid molecules. They can act as carriers that selectively deliver cytotoxic agents to hormone-dependent tumor cells resulting in improved target selectivity, diminished toxicity, and increased efficacy. Research on hybrid ligands coupling alkylating agents to steroids in the late 1960s led to discovery of estramustine phosphate, an approved anti-prostate cancer drug [17]. In the latter drug conjugate, a nitrogen mustard normustine is directly connected to a phosphate prodrug of estradiol through a labile carbamate linkage (Figure 1). A review by Dao and Hanson critically summarized advances in the field of targeting the estrogen receptor by anticancer steroid–drug conjugates till 2012 [18]. For drug conjugates incorporating more structurally diverse estrogen receptor ligands reported before 2009, the reader is referred to an excellent review “Targeting Tumors Using Estrogen Receptor Ligand Conjugates” by Keely and Meegan [19]. Here, we discuss developments in the field reported in the last decade (2010–2022). In the following chapters, the structures and pharmacological effects of drug conjugates combining tamoxifen, its active metabolites 4-OH-tamoxifen/endoxifen, and various steroidal ER-ligands with other anticancer pharmacophores are discussed and critically reviewed.

## 2. Hybrid Ligands Incorporating Tamoxifen

**Figure 2:**
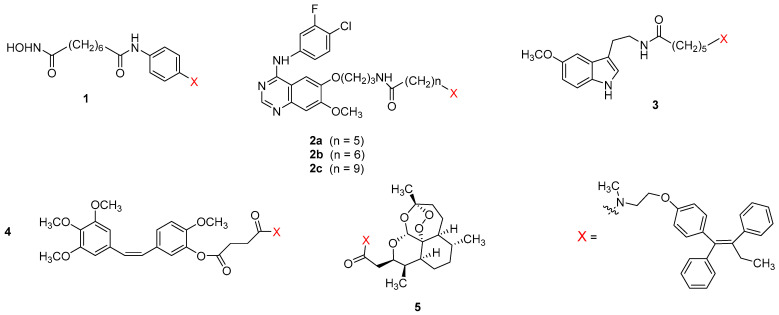
Structures of selected drug conjugates incorporating tamoxifen.

Tamoxifen belongs to the most common endocrine therapies against ER+ breast cancer. However, its chronic use can increase the risk of uterine cancer [20] and induce tamoxifen resistance [21]. Several studies indicated that particular anticancer agents including the EGFR inhibitor gefitinib [22], the HDAC inhibitor vorinostat [23], and the neurohormone melatonin [24,25,26,27], may reverse acquired resistance to tamoxifen making a possible combination therapy more effective compared to single treatment with tamoxifen. These reports prompted us and others to develop three series of hybrid ligands combining tamoxifen with vorinostat, gefitinib, and melatonin. The structures of the respective drug conjugates **1**, **2a–c** and **3** that showed the most favorable pharmacological profile are shown in Figure 2, their pharmacological data in Table 1. Additionally, drug conjugates **4** and **5** combining tamoxifen with the antimitotic and tubulin targeting agent combretastatin and antimalarial drug artemisinin, respectively are included in Figure 2 and Table 1.

**Figure 3:**
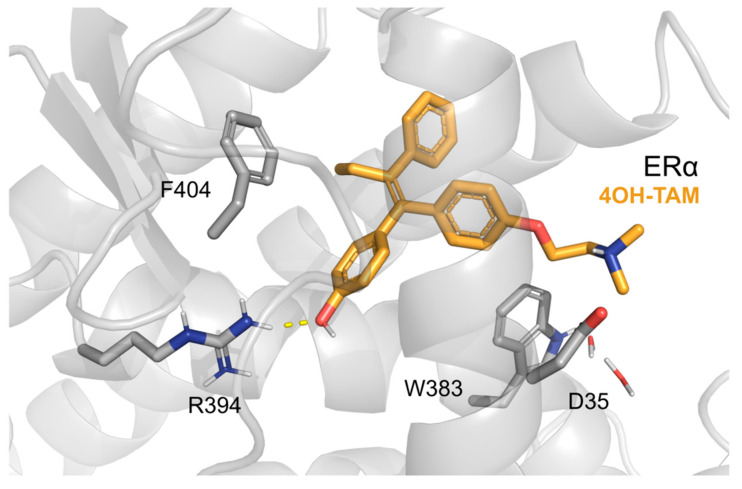
Crystal structure of 4-hydroxytamoxifen bound to ERα (PDB 3ERT). The dimethylamino group is solvent exposed allowing for attaching the linker without substantial disruption of binding.

To guarantee dual anticancer action, linking the second anticancer pharmacophore to tamoxifen must retain substantial binding to ER. In all hybrid ligands shown in Figure 2, the linker connecting tamoxifen with the second drug is attached to its aminoalkyl side chain. The dimethylamino group of tamoxifen is expected to be solvent exposed similar to that of its active metabolite 4-hydroxytamoxifen (see crystal structure of 4-hydroxytamoxifen bound to the ligand binding domain of ERα, PDB 3ERT, Figure 3), and thus, allows attaching the linker without substantial disruption of binding. Interestingly, both *N*-alkyl (hybrids **1–3**) and *N*-amide (hybrids **4–5**) linkage are well tolerated by ERα.

The vorinostat-tamoxifen hybrid **1** is not superior to the parent drugs in its antagonist action at ERα (3-fold less potent than tamoxifen) and, even stronger so, in HDAC1 and HDAC6 inhibition (65-fold and 5-fold less potent than vorinostat, respectively) [28]. However, the antiproliferative activity of **1** on ERα-positive MCF-7 breast cancer cells was 4-fold higher than for tamoxifen (EC_50_ ≈ 16 µM) and comparable to that of vorinostat (EC_50_ ≈ 4 µM for both compounds). As for growth inhibition of triple-negative MDA-MB-231 breast cancer cells, **1** (EC_50_ = 8 µM) was 2-fold more potent than tamoxifen and 2-fold less potent than vorinostat.

In a series of gefitinib-tamoxifen hybrids exemplified by compounds **2a–c** [29], the ether oxygen at C6 of gefitinib was chosen as an attachment point for the linker as the crystal structures of the gefitinib-EGFR complexes (PDB codes 3UG2 and 4WKQ) revealed the *O*-morpholinopropyl group at C6 to extend outside the ATP binding pocket toward the solvent. Among the hybrid ligands incorporating an amide (CH_2_)_n_NHCO-linker (*n* = 4–6, 9, 15), a clear correlation between the length of the polymethylene chain and the antagonist activity at ERα could be observed with the (CH_2_)_6_-linked analog **2b** showing the highest and 5-fold greater potency than tamoxifen (EC_50_ = 11 nM and 49 nM, respectively). Interestingly, compound **2b** retained strong EGFR inhibition showing 16-fold higher potency than the reference pan-kinase inhibitor staurosporine (IC_50_ = 1 nM and 16 nM, respectively) and only approximately 10-fold lower potency than gefitinib. The effect of compounds **2a** and **2b** on cell viability in MCF-7, MDA-MB-231, MDA-MB-468 and BT-549 breast cancer cells will be discussed in the following chapter together with the effect of the hydroxylated analogs **6a** and **6b** in which the tamoxifen pharmacophore has been replaced by its active metabolite 4-hydroxytamoxifen.

A series of hybrid ligands incorporating tamoxifen connected to the neurohormone melatonin through the side chain of the latter has been reported in the patent literature [30]. Detailed pharmacological data have been only reported for the (CH_2_)_5_-linked analog **3**. In competition binding experiments on ERα expressed in mouse uterus using [^125^I]-estradiol, compound **3** showed 5-fold higher affinity toward ERα than tamoxifen (IC_50_ = 2.2 nM vs. 10 nM, respectively). As for binding to melatonin MT_1_ receptors that are thought to be responsible for melatonin’s anti-cancer actions, the hybrid ligand **3** was also superior to the parent drug showing 3-fold higher affinity in [^125^I]-iodomelatonin competition binding experiments at human MT_1_ receptors expressed in CHO cells (2.8 nM vs. 8.6 nM, respectively). The higher MT_1_-affinity of compound **3** compared to the parent drug is rather surprising taking into account that according to the well-established SARs, alkyl substituents larger than propyl attached to the amide carbonyl group of melatonin reduce binding affinity to melatonin receptors [33,34]. Most importantly, in vivo studies in mice showed that hybrid ligand **3** created similar anticancer effects in mammary tissue as tamoxifen, but did not create hyperproliferation of uterine tissue that was caused by tamoxifen alone or by the combination of melatonin and tamoxifen. A later publication reported a full pharmacological characterization of compound **3** and related hybrid ligands with (CH_2_)n-linkers of different lengths (*n* = 2, 4, 9, 15) including their effects on viability and migration of MCF-7, MMC, MDA-MB-231, BT-549 and tamoxifen-resistant MCF-7 breast cancer cells and identification of the signaling proteins/cascades involved [35].

A conjugate molecule **4** incorporating tamoxifen connected to an antimitotic and tubulin targeting agent combretastatin A-4 through a succinic acid ester linkage was reported to display comparable binding affinity to ERα to that of endoxifen, the active metabolite of tamoxifen (IC_50_ = 80 nM and 47 nM, respectively) [31]. As for antiproliferative activity at MCF-7 cells, compound **4** was 23-fold more potent than tamoxifen (IC_50_ = 90 nM vs. 2.13 µM) and 11-fold less potent than combretastatin (IC_50_ = 8 nM). Further screening on 60 cancer cell lines of diverse tumor origin including colon cancer (HCC-2998, HCT-116, HCT-15, breast cancer (BT-549, MCF-7, MDA-MB-468), melanoma (M14) and CNS cancer (SF-295) revealed very potent growth inhibition with GI_50_ values within the range 10–72 nM. For MCF-7 cells, a large therapeutic window was found between the concentration required for inhibition of cancer cell growth (GI_50_ = 50 nM) and the one determined to be toxic (LD_50_ >10 µM). As hybrid ligand **4** showed no selectivity towards the ER-positive MCF-7 cell line, its antiproliferative activity is most likely not related to blocking ERs but rather to the inhibition of tubulin polymerization (an effect caused by the combretastatin unit) or other biological mechanisms. However, it remains unclear if compound **4** undergoes enzymatical ester hydrolysis before reaching its anticancer targets, and consequently if its anticancer actions are not mainly caused by liberated combretastatin A-4. The pharmacological properties of a structurally related drug conjugate **7** with tamoxifen pharmacophore replaced by its active metabolite 4-hydroxytamoxifen are discussed in the following chapter.

In a recently published a series of hybrid ligands of tamoxifen and the antimalarial drug artemisinin, the most potent drug conjugate (compound **5**) showed low micromolar growth inhibition (EC_50_ = 4 µM) and was approximately 5-fold more potent than artesunic acid and *E*/*Z*-endoxifen in the antiproliferation assay on MCF-7 cells. Other pharmacological data were not reported [32].

A disadvantage of tamoxifen drug conjugates as ER-ligands is their relatively low binding affinity/antagonist activity at ERα which is at best in the same concentration range as for the parent drug. However, while tamoxifen is considered a prodrug metabolized by cytochrome P-450 enzymes to considerably more potent ER-antagonists (*Z*)-4-hydro-xytamoxifen and (*Z*)-endoxifen, respectively (see Table 2), biotransformation of the tamoxifen-incorporating hybrid ligands would not necessarily generate the respective hydroxylated analogs with higher antiestrogen activity. For example, the gefitinib-tamoxifen drug conjugate **2c** was found to be metabolically very stable in mouse liver microsomes over 120 min. In contrast, the melatonin-tamoxifen hybrid **3** undergoes similar oxidative metabolism in mouse and human microsomes as tamoxifen but the structures and pharmacological effects of the metabolites were not determined.

In order to ensure high ER-binding, most research groups incorporated 4-hydroxytamoxifen/endoxifen into their hybrid ligands instead of, or in addition to, tamoxifen. A selection of the corresponding drug conjugates is presented in the next chapter. As their structures exclusively include tertiary amino groups, we refer to these compounds as conjugates of 4-hydroxytamoxifen (4-OH-tamoxifen) rather than of endoxifen that is a secondary amine.

## 3. Hybrid Ligands Incorporating 4-Hydroxytamoxifen

As mentioned in the previous chapter, the rationale for incorporating 4-OH-tamoxifen in anticancer hybrid ligands is its higher ER-binding and antagonist potency compared to tamoxifen (see Table 2). Interestingly, the more pronounced antiestrogen activity of 4-OH-tamoxifen correlates with its higher growth inhibition of ER-positive MCF-7 breast cancer cells. In particular, compared to tamoxifen, 4-OH-tamoxifen showed a 100-fold higher ability to prevent MCF-7 cell growth as a consequence of its 300-fold higher affinity for ER (determined by binding competition with [^3^H]-estradiol on the uterine and MCF7 cytosol ER) [38].

The pharmacological action of tamoxifen and 4-OH-tamoxifen is strongly dependent on the isomeric state of their central double bonds with the (*Z*)-isomers (formerly known as *trans*) possessing antiestrogen activity and, on the contrary, (*E)*-isomers (formerly known as *cis*) being weak estrogens [39]. Consequently, tamoxifen is marketed as a single (*Z*)-stereoisomer. While tamoxifen undergoes no double-bond isomerization to (*E*)-tamoxifen under physiological conditions, (*Z*)-4-OH-tamoxifen can be interconverted to the corresponding (*E*)-isomer in vitro [40], probably because of the electron-donating effect of the hydroxy group that stabilizes by resonance the intermediate carbocation obtained by initial protonation of the double bond. For example, 20% of (*Z*)-4-OH-tamoxifen was reported to have isomerized to the corresponding (*E*)-isomer after 2 days at 37 °C in tissue culture medium including estrogen receptor-positive MCF-7 human breast cancer cells [41]. Interestingly, from both isomers present, the MCF-7 cells preferentially accumulated the (Z)-isomer and the material associated with the nuclear estrogen receptor contained mainly the higher affinity (*Z*)-isomer [40].

**Figure 4:**
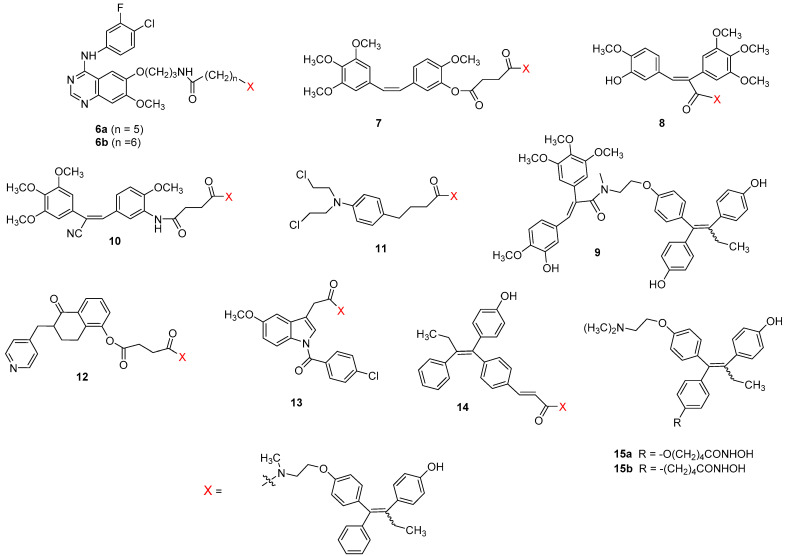
Structures of selected drug conjugates incorporating 4-hydroxytamoxifen.

The structures of the drug conjugates incorporating 4-OH-tamoxifen with the most favorable pharmacological profile (**6–14**) are shown in Figure 4 and their pharmacological data in Table 3.

The majority of these hybrid ligands are mixtures of (*Z*) and (*E*)-isomers, mostly in a 1:1 ratio. The only exception are the gefitinib-4-OH-tamoxifen hybrids **6a** and **6b** (Figure 4) that have been synthesized and pharmacologically tested as potentially more active pure (*Z*)-isomers.

The hybrid ligand **6b** is a direct analog of the tamoxifen-gefitinib drug conjugate **2b** (Table 1) with the tamoxifen part replaced by (*Z*)-4-OH-tamoxifen [29]. As expected, compound **6b** is a significantly more potent ERα-antagonist than compound **2** (EC_50_ = 4.4 nM vs. 11nM) although the increase in antiestrogen activity is less pronounced than for the parent drugs tamoxifen and (*Z*)-4-OH-tamoxifen (EC_50_ = 49 nM vs. 0.21 nM). Drug conjugate **6a** with a shorter linker than in **6b** (five CH_2_-groups vs. six CH_2_-groups) is equipotent to **6b** at ERα but is surprisingly 100-fold more potent as EGFR inhibitor (EC_50_ = 2.5 nM vs. 260 nM) making **6a** a more promising anti-cancer agent. However, **6a** and **6b** are nearly equipotent in inhibiting cell viability of MCF-7 (IC_50_ = 1.35 µM vs. 2.00 µM), MDA-MB-231 (IC_50_ = 890 nM vs. 970 nM), MDA-MB-468 (IC_50_ = 550nM vs. 490 nM) and BT-549 (IC_50_ = 460 nM vs. 380 nM) cells after 5 days of treatment as determined in an XTT assay. Moreover, in a crystal violet assay, these compounds at 2 µM concentrations showed higher or comparable inhibition of all four types of breast cancer cells compared to 5 µM of endoxifen alone, gefitinib alone or combinatorial treatment with endoxifen/gefitinib at 5 µM each. Interestingly, treatment of MCF-7 cells with saturating 2 µM concentration of **6a** and **6b** led to reduced expression of ERα indicating their possible action as SERDs. An ER-independent mechanism of action is also supported by their nanomolar IC_50_-values (380–970 nM) in all three types of triple-negative breast cancer cells (MDA-MB-231, MDA-MB-468 and BT-549 and by very similar IC_50_-values of the respective non-hydroxylated analogs **2a** and **2b** in all cancer cell lines including ER-positive MCF-7 cells. Metabolic studies of **6a** and **6b** using mouse liver microsomes demonstrated only slight (<10%) degradation indicating that the amide linkage is metabolically stable. Moreover, ADME predictions suggested that despite large molecular weights these compounds are expected to be orally bioavailable.

The combrestatine hybrid ligand **7** is a direct analog of compound **4** with the tamoxifen pharmacophore replaced by (*Z*/*E*)-4-OH-tamoxifen. It displayed nanomolar binding to ERα (IC_50_ = 52 nM) being slightly higher than that of **4** (IC_50_ = 80 nM) and only slightly lower than for (*Z*/*E*)-4-OH-tamoxifen (IC_50_ = 30 nM). As for antiproliferative activity at MCF-7 cells, compound **7** showed extraordinarily high potency (IC_50_ = 5.7 nM) significantly higher than for both parent drugs combretastatin and (*Z*/*E*)-endoxifen (IC_50_ = 8 nM and 29 nM) and 15-fold higher compared to compound **4**. However, similar to compound **4**, the metabolic stability of compound **7**, in particular, related to potential ester hydrolysis that would lead to a mixture of unliked combretastatin and *N*-succinamide-substituted (*Z*/*E*)-4-OH-tamoxifen was not reported.

Another series of drug conjugate connecting a double bond of combretastatin with (*Z*/*E*)-4-OH-tamoxifen through a direct acrylamide linkage was reported by the same research group revealing compound **8** as the most potent analog with respect to its anti-proliferative action on human MCF-7 cells (IC_50_ = 33 nM) [42]. Interestingly, *para*-hydro-xylation of the second aromatic ring resulted in a 6-fold more potent analog **9** (IC_50_ = 5 nM). The potent antiproliferative action of compound **9** seems to be mediated by its antiestrogenic effect as this drug conjugate showed an extraordinarily high affinity towards ERα (IC_50_ = 0.9 nM) being 30–50 fold higher than those of the control drugs (*Z*/*E*)-4-OH-tamoxifen, (*Z*/*E*)-endoxifen and (*Z*/*E*)-hydroxyendoxifen (IC_50_ = 30 nM, 47 nM and 44 nM, respectively) and even 6-fold higher than that of estradiol (IC_50_ = 6 nM). As for antiproliferative activity in the ER-negative MDA-MB-231 human breast cancer cells, drug conjugates **8** and **9** displayed, as expected, only micromolar IC_50_-values (2.67 µM and 2.48 µM) being 8-fold more potent than tamoxifen and (*Z*/*E*)-4-OH-tamoxifen (20 µM and 18 µM). Comparison of the IC_50_-values obtained on MCF-7 cells (ER-positive) to those from MDA-MB-231 cells (ER-negative) identifies antiestrogenic action of **8** and **9** as a mechanism contributing to their strong growth inhibition of breast cancer cells.

In the hybrid ligand **10**, (*Z*/*E*)-4-OH-tamoxifen is attached to an aromatic ring of cyano-combretastatin using a succinamide linkage [43]. Compound **10** showed only moderate binding to ERα (IC_50_ = 254 nM) and moderate antiproliferative activity (IC_50_ = 1.64 µM) in MCF-7 cells.

Among drug conjugates **11–13** linking (*Z*/*E*)-4-OH-tamoxifen with the alkylating agent chlorambucil (**11**), a tetralone aromatase inhibitor (**12**) and a COX inhibitor indomethacin (**13**), the hybrid ligands **11** and **12** displayed similar affinity toward ERα (IC_50_ = 51 nM and 79 nM) as tamoxifen (IC_50_ = 70 nM), while compound **11** showed weaker binding (IC_50_ = 524 nM). All three compounds were only moderately potent in their antiproliferative action on MCF-7 cells (IC_50_ = 12–30 µM) [43].

In the hybrid ligand **14**, (*Z*/*E*)-4-OH-tamoxifen is connected to another 4-hydroxylated analog of tamoxifen through an acrylamide linkage. Despite higher affinity toward ERα than tamoxifen (IC_50_ = 36 nM vs. 70 nM), compound **9** exhibited only very low antiproliferative effect on MCF-7 cells (IC_50_ > 50 µM).

Hybrid ligands **15a** and **15b** are (*Z*/*E*)-4-OH-tamoxifen analogs with the phenyl group substituted in the *para* position with the Zn-chelating hydroxamic acids -(CH_2_)_4_CONHOH and -O(CH_2_)_4_CONHOH, respectively, that are structurally related to the HDAC inhibitor vorinostat [44]. Both hybrids **15a** and **15b** (IC_50_ = 800 nM), retained the antiestrogenic activity of the parent drug at ERα (IC_50_ = 500 nM) but were significantly less potent HDAC6 (**15a**: 30-fold, **15b**: 6-fold) and HDAC1 (**15a**: 20-fold, **15b**: 7-fold) inhibitors than vorinostat. Compound **15b** displayed antiproliferative action on MCF-7 cells in the low micromolar concentration and was superior to both parent drugs. In the triple-negative MDA-MB-231 cells and ER-negative MCF-10A cells, **15b** showed cytotoxicity in the micromolar range with an IC_50_ intermediate between that of vorinostat and 4-OH-tamoxifen.

## 4. Hybrid Ligands Incorporating Steroids

**Figure 5:**
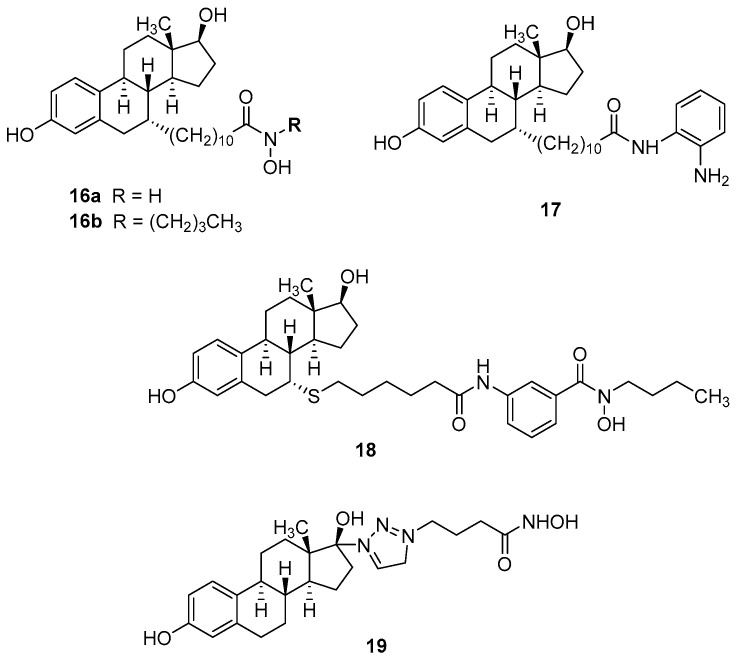
Structures of selected drug conjugates incorporating steroids.

Hybrid ligands combining the pure antiestrogen and SERD ICI-164384 (Figure 1) with the HDAC inhibitors structurally related to vorinostat (**16a** and **16b**) and entinostat (**17**) and a benzhydroxamic acid analog **18** were reported [45]. Their structures and pharmacological data are shown in Figure 5 and Table 4. The hybrid ligand **18** demonstrated the most favorable pharmacological profile with only 3-fold lower antagonist action at ERα (IC_50_ = 180 nM) than the parent SERD drug ICI-164384 (IC_50_ = 50 nM) and, most likely based on the additional HDAC inhibition, 3-fold higher antiproliferative activity on MCF-7 cells. The ER-negative MDA-MB-231 cells were inhibited by compound **18** within one order of magnitude of that of vorinostat.

ER agonists were rarely incorporated into anticancer hybrid ligands. To the best of our knowledge, only one series of estradiol-incorporating drug conjugates, namely vorinostat analogs, covalently attached to C-17 of estradiol through triazole-polymethylene linkers have been reported since 2012. The triazole-(CH_2_)_3_-linked analog **19** demonstrated the best pharmacological profile showing 4-fold higher HDAC6 inhibition than vorinostat (IC_50_ = 8 nM vs. 34 nM) and only moderately lower antiproliferative action than the parent drug at both MCF-7 (IC_50_ = 22 µM vs. 4.4 µM) and MDA-MB-231 cells (IC_50_ = 28 µM vs. 3.4 µM).

## 5. Summary and Conclusions

Numerous drug conjugates incorporating estrogen receptor ligands were reported in the last decade. Most of the hybrids are derivatives of tamoxifen and of its much more active metabolites 4-OH-tamoxifen/endoxifen with the latter analogs showing, as expected, higher antiestrogenic activity. The most potent ERα-antagonists are (*Z*)-4-OH-tamoxifen drug conjugates incorporating the EGFR inhibitor gefitinib (**6a**, **6b**) and the antimitotic agent combretastatin A-4 (**9**) displaying single-digit nanomolar IC_50_ values. While compound **9** was also the most potent hybrid ligand in the antiproliferative assay at ER-positive MCF-7 cells indicating that its anticancer action is mediated through binding to ERs, the gefitinib hybrids **6a** and **6b** were more potent at triple-negative breast cancer cells suggesting an ER-independent mechanism of action.

Whereas hybrid ligands combining antiestrogens with other classes of anticancer drugs represent without doubt valuable pharmacological tools and can be considered as an attempt to carrier drug delivery (estrogen receptor pharmacophore “delivers” the hybrids to cancer cells overexpressing estrogen receptors), a question remains whether they are superior to a combination of both single drugs in a clinical setting.

One potential advantage of hybrid ligands could be controlled toxicity. Drugs within combinations are often given in maximum-tolerated doses, which means that it is not the efficacy at the site of action that determines the dose, but, rather, the overall additive toxicity. However, when the active concentrations at the two anticancer targets considerably differ (e.g., nanomolar activity at ER and micromolar at the second target), the determination of the effective and safe dose would be difficult for hybrid drugs as they include both pharmacophores in a fixed 1:1 ratio. Moreover, attachment of the linker and the second anti-cancer pharmacophore usually results in reduced activity at both targets, and consequently higher effective doses compared to single drugs increasing the risk of toxic side effects. The latter problem could be possibly tackled by using chemically labile linkers that would ideally enable a controlled, tumor-specific cleavage reaction, releasing active drugs at the site of action with sufficient stability in the bloodstream to avoid systemic toxicity, a strategy that has been followed in the carbamate-linked estramustine phosphate. However, in the drug conjugates reported here, no cleavable linkers have been used except esters that are very likely to be hydrolyzed before reaching the target tumors.

Another often claimed potential advantage of hybrid ligands compared to a combination of single-target drugs is that while two single drugs show different pharmacokinetics reaching the target tumors not simultaneously, a hybrid drug is a single chemical entity delivering the two pharmacophores to their anticancer targets at the same time enabling the optimal synergistic anticancer effect. While this might be true when both molecular targets are intracellular (e.g., nuclear receptors, histone deacetylases, DNA, etc.), a simultaneous binding to membrane receptors (e.g., melatonin receptors) and intracellular targets seems very unlikely and, given the short length of the spacers, this binding would only be possible when different hybrid ligand molecules are involved, where one molecule binds to the extra-cellular binding site of the membrane receptor, while the other must pass the cell membrane in order to bind to intracellular targets.

A challenge for the design and development of drug conjugates as anticancer therapeutics is the optimization of their physicochemical properties towards a favorable pharmacokinetic profile [46]. Especially the hybrid ligands with the pharmacophores connected by longer spacers are large chemical entities with molecular mass exceeding 500 Da and often high lipophilicity and violate Lipinski’s [47] and Veber’s [48] rules that assess molecular properties influencing oral bioavailability. One strategy to avoid higher molecular weight is to merge two pharmacophores into a more compact, smaller drug conjugate. Notably, in most anticancer drug conjugates currently undergoing clinical trials, the pharmacophores are not connected by a linker, but rather fused into one compact molecule resulting in relatively low molecular weights. Recent examples are CUDC-101 (MW 434), a dually acting chimeric EGFR/HDAC inhibitor [7,8]; fimepinostat (CUDC-907, MW 509), a dual HDAC-PI3K inhibitor [9]; tinostamustine (MW 415), a fusion molecule composed of an alkylating agent bendamustine and HDAC inhibitor vorinostat [49]. As for the drug conjugates presented in this review, the “physichochemical challenge” has not been addressed. Moreover, for the majority of the compounds, their metabolic stability and/or activation (e.g., through 4-hydroxylation of the tamoxifen part) and in vivo studies were not reported making their assessment as potential drugs difficult.

Lastly, a very recent “expert opinion” review entitled “Have molecular hybrids delivered effective anti-cancer treatments and what should future drug discovery focus on?” describes a rather optimistic picture of anticancer molecular hybrids in terms of current and future anticancer treatment [50]. Admittedly, there has been an increased interest for anticancer drug conjugates in the last decade, especially in academia, as indicated by the rising number of publications in the field. However, as shown in this review for drug conjugates incorporating ER ligands, there is still a long way to go until molecular hybridization becomes a widely established principle in the design of anticancer drugs.

## Figures and Tables

**Table 1:** Pharmacological actions of drug conjugates incorporating tamoxifen.

	Activity at ERα	Antiproliferative Activity		Activity at Second Target	Lit.
	IC_50_	MCF-7	MDA-MB-231		
1	127 nM ^a^	3.8 µM	8.1 µM	HDAC1 2.7 µM	[28]
				HDAC6 221 nM	
tamoxifen	39 nM ^a^	16 µM	17 µM	-	
vorinostat	-	4.4 µM	3.4 µM	HDAC1 42 nM	
				HDAC6 34 nM	
**2a**	101 nM ^a^	1.2 µM	780 nM	-	[29]
**2b**	11 nM ^a^	1.5 µM	850 nM	EGFR 1.1 nM	
**2c**	232 nM ^a^	-	-	-	
tamoxifen	49 nM ^a^				
gefitinib	-	-	-	EGFR < 0.1 nM	
**3**	2 nM ^b^	-	-	MT_1_ 2.8 nM ^c^	[30]
tamoxifen	10 nM ^b^	-	-	^-^	
melatonin	-	-	-	MT_1_ 8.6 nM ^c^	
**4**	80 nM ^d^	90 nM	-	-	[31]
tamoxifen	-	2.1 µM	-	-	
combretastatin	-	8 nM	-		
**5**	-	3.9 µM	-	-	[32]
artesunic acid	-	32 µM		-	

^a^ Antagonist activity in ERα luciferase reporter assay, ^b^ Competition with [^125^I]-estradiol for binding to ERs expressed in mouse uterus, ^c^ Competition with 2-[^125^I]-melatonin for binding to melatonin MT_1_ receptors, ^d^ binding affinity in fluorescence polarization based competitive binding assay.

**Table 2:** Structures of major metabolites of tamoxifen, their plasma concentrations in women taking tamoxifen [36], their antagonist activities at ERα [29] and binding affinities to rat uterine ERs [37].

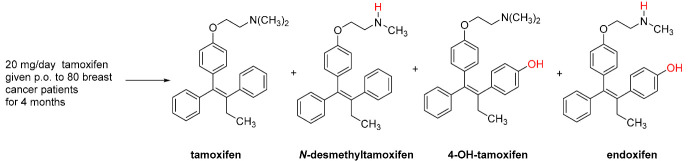				
plasma concentration	363 nM	655 nM	9 nM	63 nM
ERα antagonist action from luciferase reporter assay IC_50_	49 nM	-	0.21 nM	0.14 nM
Inhibition of [^3^H]-estradiol binding to rat uterine ERs IC_50_	~100 nM	~200 nM	~3 nM	-

**Table 3:** Pharmacological actions of drug conjugates incorporating 4-hydroxytamoxifen.

	Activity at ERα	Antiproliferative Activity		Activity at Second Target	Lit.
	IC_50_	MCF-7	MDA-MB-231		
**6a**	4.6 nM ^a^	1.4 µM	890 nM	EGFR 2.5 nM	[29]
**6b**	4.4 nM ^a^	2.0 µM	970 nM	EGFR 260 nM	
*Z*-4-OH-tamoxifen	0.21 nM ^a^	-	-	-	
*Z*-endoxifen	0.14 nM ^a^	-	-	-	
gefitinib	-			EGFR < 0.1 nM	
**7**	52 nM ^b^	5.7 nM	-		[31]
**8**	-	33 nM	2.7 µM		[42]
**9**	1 nM ^b^	5 nM	2.5 µM		
E/Z-endoxifen	47 nM ^b^	29 nM	-		
4-OH-tamoxifen	30 nM ^b^	-	18 µM		
tamoxifen	-	-	20 µM		
combretastatin	-	8 nM	43 nM		
**10**	524 nM	1.6 µM			[43]
**11**	490 nM	30 µM			
**12**	51 nM	13 µM			
**13**	79 nM	12 µM			
**14**	36 nM	>50 µM			
tamoxifen	70 nM	4 µM			
**15a**	800 nM ^c^	1.2 µM		HDAC6 1.78 µM	[44]
**15b**	820 nM ^c^	790 nM	1.1 µM	HDAC3 2.10 µM	
				HDAC6 0.30 µM	
				HDAC3 0.73 µM	
4-OH-tamoxifen	500 nM ^c^	3.3 µM	2.5 µM	HDAC6 0.06 µM	
vorinostat	-	450 nM	610 nM		
				HDAC3 0.11 µM	

^a^ Antagonist activity in ERα luciferase reporter assay, ^b^ binding affinity in fluorescence polarization based competitive binding assay, ^c^ Antagonist activity in BRET assay.

**Table 4:** Pharmacological actions of selected drug conjugates incorporating steroids.

	Activity at ERα IC_50_^a^	Antiproliferative Activity MCF-7 Cells	HDAC6 Inhibition	HDAC3 Inhibition	Lit
**16a**	1.06 µM	2.93 µM	1.15 µM	960 nM	[45]
**16b**	2.10 µM	9.11 µM	>50 µM	>5 µM	
**17**	720 nM	1.90 µM	>50 µM	3.18 µM	
**18**	180 nM	340 nM	43.7 µM	1.55 µM	
*Z*-4-OH-tamoxifen	10 nM	150 nM	-	-	
ICI-164384	50 nM	930 nM	-	-	
vorinostat	-	320 nM	350 nM	170 nM	
entinostat	-	350 nM	-	310 nM	
**19**	22 µM	22 µM	8 nM	-	[28]
estradiol	-	-	-	-	
vorinostat	4.4 µM	4.4 µM	34 nM	-	

^a^ Antagonist activity in ERα luciferase reporter assay.

## Data Availability

Not applicable.
